# Supplementary material for: Plasma Lysophosphatidic Acid Concentrations in Sex Differences and Psychiatric Comorbidity in Patients with Cocaine Use Disorder
Source: Int J Mol Sci. 2023 Oct 25;24(21):15586. doi: 10.3390/ijms242115586 (PMC10649657; doi:10.3390/ijms242115586)
Supplement: Supplementary file 1 [file ijms-24-15586-s001.zip › ijms-2657060-supplementary.pdf]

Table S1: Plasma concentrations of LPA species grouped according to history of CUD.

Variables	Control group (N=60)	CUD group (N=88)	Statistics[1]		
	Mean [95%CI]	Mean [95%CI]	F-value	df	*p*-value
**Total LPA (ng/mL)**	142.89 [127.64-159.58]	149.27 [136.14-164.05]	0.39	1.142	0.534
**16:0 LPA (ng/mL)**	21.78 [19.63-24.15]	24.49 [22.49-26.73]	3.08	1.142	0.081
**18:0 LPA (ng/mL)**	8.60 [7.41-10]	10.16 [8.97-11.53]	2.85	1.142	0.093
**18:1 LPA (ng/mL)**	11.97 [10.71-13.38]	10.96 [9.98-12.05]	1.43	1.142	0.233
**18:2 LPA (ng/mL)**	69.50 [60.67-79.79]	66.68 [59.43-74.82]	0.22	1.142	0.644
**20:4 LPA (ng/mL)**	28.51 [25.17-32.28]	31.99 [28.77-35.48]	0.05	1.142	0.165

[1]*Two-way ANCOVA was performed using lifetime CUD and sex as factors and controlling for age and BMI as covariates. Abbreviations: LPA= lysophosphatidic acid; df = degree of freedom; CUD= cocaine use disorder.*

Table S2: Plasma concentrations of LPA species grouped according to sex in both control subjects and CUD patients.

Variables	Men (N=124)	Women (N=24)	Statistics[1]		
	Mean [95%CI]	Mean [95%CI]	F-value	df	*p*-value
**Total LPA (ng/mL)**	127.79 [120.50-143.75]	166.72 [145.88-190.99]	12.84	1.142	**<0.001**
**16:0 LPA (ng/mL)**	19.95 [18.92-21.08]	26.72 [23.60-30.26]	17.76	1.142	**<0.001**
**18:0 LPA (ng/mL)**	9.37 [8.66-10.13]	9.35 [7.80-11.22]	0.00	1.142	0.986
**18:1 LPA (ng/mL)**	10.28 [9.71-10.91]	12.76 [11.17-14.59]	8.39	1.142	**0.004**
**18:2 LPA (ng/mL)**	58.21 [54.20-62.66]	79.61 [67.45-93.97]	11.59	1.142	**0.001**
**20:4 LPA (ng/mL)**	28.65 [25.74-31.56]	39.43 [32.76-46.11]	6.945	1.142	**0.009**

[1]Two-*way ANCOVA was performed using lifetime CUD and sex as factors and controlling for age and BMI as covariates. Bold values are statistically significant for p<0.05. Abbreviations: LPA= lysophosphatidic acid; df = degree of freedom.*

Table S3: Plasma concentrations of LPA species grouped according to history of mood disorder in CUD patients.

Variables	No mood disorder (N=62)	Comorbid mood disorder (N=26)	Statistics[1]		
	Mean [95%CI]	Mean [95%CI]	F-value	df	*p*-value
**Total LPA (ng/mL)**	153.46 [137.40-171.39]	137.40 [112.20-168.26]	0.86	1.88	0.358
**16:0 LPA (ng/mL)**	25.17 [24.38-30.25]	22.80 [18.62-27.93]	0.711	1.88	0.402
**18:0 LPA (ng/mL)**	10.57 [8.99-12.45]	8.81 [6.55-11.89]	1.09	1.88	0.079
**18:1 LPA (ng/mL)**	11.28 [10.16-12.53]	9.79 [8.07-11.91]	1.54	1.88	0.218
**18:2 LPA (ng/mL)**	67.68 [58.88-77.80]	64.86 [50.23-83.75]	0.08	1.88	0.779
**20:4 LPA (ng/mL)**	33.72 [29.85-38.11]	26.48 [21.13-33.11]	3.41	1.88	0.068

[1]*Two-way ANCOVA was performed using lifetime CUD and sex as factors and controlling for age and BMI as covariates. Abbreviations: LPA= lysophosphatidic acid; df = degree of freedom.*

Table S4: Plasma concentrations of LPA species grouped according to history of anxiety disorder in CUD patients.

Variables	No anxiety disorder (N=65)	Comorbid anxiety disorder (N=23)	Statistics[1]		
	Mean [95%CI]	Mean [95%CI]	F-value	df	*p*-value
**Total LPA (ng/mL)**	173.78 [149.97-201.84]	135.21 [118.30-154.88]	6.03	1.88	**0.016**
**16:0 LPA (ng/mL)**	26.79 [23.01-31.19]	22.85 [19.95-26.24]	2.30	1.88	0.133
**18:0 LPA (ng/mL)**	12.41 [9.93-15.52]	8.61 [7.03-10.54]	5.75	1.88	**0.019**
**18:1 LPA (ng/mL)**	11.72 [10.11-13.58]	10.62 [9.29-12.13]	1.68	1.88	0.199
**18:2 LPA (ng/mL)**	76.91 [63.68-93.11]	61.23 [51.52-72.78]	3.06	1.88	0.084
**20:4 LPA (ng/mL)**	37.32 [31.55-44.05]	29.17 [25.06-33.96]	0.11	1.88	**0.035**

[1]*Two-way ANCOVA was performed using lifetime CUD and sex as factors and controlling for age and BMI as covariates. Bold values are statistically significant for p<0.05. Abbreviations: LPA= lysophosphatidic acid; df = degree of freedom.*

Table S5: Plasma concentrations of LPA species grouped according to history of personality disorder in CUD patients.

VARIABLE	No personality disorder (N=68)	Comorbid personality disorder (N=20)	Statistics[1]		
	Mean [95%CI]	Mean [95%CI]	F-value	df	*p*-value
**Total LPA (ng/mL)**	144.54 [129.42-161.06]	164.82 [137.72-197.24]	1.55	1.88	0.217
**16:0 LPA (ng/mL)**	23.88 [21.43-26.67]	26.61 [22.23-31.84]	1.03	1.88	0.313
**18:0 LPA (ng/mL)**	10.07 [8.53-11.86]	10.66 [8.09-13.90]	0.11	1.88	0.714
**18:1 LPA (ng/mL)**	10.28 [9.27-11.38]	12.91 [10.91-15.28]	5.31	1.88	**0.024**
**18:2 LPA (ng/mL)**	67.14 [58.48-77.09]	66.68 [53.21-83.56]	0.00	1.88	0.952
**20:4 LPA (ng/mL)**	29.04 [25.76-32.66]	40.55 [33.41-49.32]	8.54	1.88	**0.004**

[1]Two-way ANCOVA was performed using lifetime CUD and sex as factors and controlling for age and BMI as covariates. Bold values are statistically significant for p<0.05. Abbreviations: LPA= lysophosphatidic acid; df = degree of freedom.

Table S6: Plasma concentrations of LPA species grouped according to history of childhood ADH disorder in CUD patients.

**Variables**	**No ADH (N=72)**	**Comorbid ADH disorder (N=16)**	**Statistics[1]**		
	**Mean [95%CI]**	**Mean [95%CI]**	**F-value**	**df**	***p*-value**
**Total LPA (ng/mL)**	154.52 [140.28-170.22]	105.92 [76.56-146.56]	4.93	1.88	**0.029**
**16:0 LPA (ng/mL)**	25.64 [23.33-28.18]	17.02 [12.39-23.39]	6.07	1.88	**0.016**
**18:0 LPA (ng/mL)**	10.28 [8.87-11.94]	8.65 [5.27-14.22]	0.44	1.88	0.508
**18:1 LPA (ng/mL)**	11.11 [10.12-12.25]	9.06 [6.59-12.45]	1.52	1.88	0.221
**18:2 LPA (ng/mL)**	69.34 [61.23-78.34]	46.34 [30.69-69.82]	3.52	1.88	0.064
**20:4 LPA (ng/mL)**	32.73 [29.30-36.56]	23.07 [15.96-33.34]	3.31	1.88	0.074

[1]*Two-way ANCOVA was performed using lifetime CUD and sex as factors and controlling for age and BMI as covariates. Bold values are statistically significant for p<0.05. Abbreviations: LPA= lysophosphatidic acid; df = degree of freedom.*

Table S7: Plasma concentrations of LPA species grouped according to comorbid alcohol disorder.

Variables	No comorbid alcohol use disorder (N=42)	Comorbid alcohol use disorder (N=46)	Statistics[1]		
	Mean [95%CI]	Mean [95%CI]	F-value	df	*p*-value
**Total LPA (ng/mL)**	151.84 [132.12-174.58]	147.23 [128.82-168.65]	0.091	1.88	0.764
**16:0 LPA (ng/mL)**	24.95 [21.72-28.71]	24.21 [21.18-27.67]	0.097	1.88	0.756
**18:0 LPA (ng/mL)**	10.69 [8.68-13.15]	9.77 [8-11.94]	0.372	1.88	0.544
**18:1 LPA (ng/mL)**	11.61 [10.19-13.27]	10.28 [9.03-11.67]	1.70	1.88	0.196
**18:2 LPA (ng/mL)**	70.30 [59.02-34.75]	63.83 [53.95-75.51]	0.621	1.88	0.433
**20:4 LPA (ng/mL)**	29.71 [25.40-1.48]	33.81 [29.04-39.26]	1.32	1.88	0.495

[1]*Two-way ANCOVA was performed using lifetime CUD and sex as factors and controlling for age and BMI as covariates. Abbreviations: LPA= lysophosphatidic acid; df = degree of freedom.*

Table S8: Plasma concentrations of LPA species grouped according to comorbid cannabis disorder in CUD patients.

Variables	No comorbid cannabis use disorder (N=27)	Comorbid cannabis use disorder (N=61)	Statistics[1]		
	Mean [95%CI]	Mean [95%CI]	F-value	df	*p*-value
**Total LPA (ng/mL)**	149.96 [135.83-165.20]	124.74 [110.15-141.25]	0.034	1.88	0.854
**16:0 LPA (ng/mL)**	24.49 [22.23-26.97]	20.94 [18.49-23.71]	0.102	1.88	0.750
**18:0 LPA (ng/mL)**	10.42 [9.02-12.05]	9.04 [7.52-10.86]	1.328	1.88	0.252
**18:1 LPA (ng/mL)**	10.93 [9.95.62-12.02]	9.59 [8.51-10.81]	0.020	1.88	0.889
**18:2 LPA (ng/mL)**	66.68 [59.02-75.51]	55.71 [47.75-65.16]	0.013	1.88	0.910
**20:4 LPA (ng/mL)**	31.77 [28.44-35.48]	26.79 [23.28-30.76]	0.003	1.88	0.975

[1]*Two-way ANCOVA was performed using lifetime CUD and sex as factors and controlling for age and BMI as covariates. Abbreviations: LPA= lysophosphatidic acid; df = degree of freedom.*

Table S9: Plasma concentrations of LPA species grouped according to comorbid medical problem in CUD patients.

Variables	No comorbid medical problem (N=67)	Comorbid medical problem (N=14)	Statistics[1]		
	Mean [95%CI]	Mean [95%CI]	F-value	df	*p*-value
**Total LPA (ng/mL)**	142.56 [125.89-161.43]	154.53 [127.35-187.93]	0.471	1.81	0.495
**16:0 LPA (ng/mL)**	24.71 [21.78-28.05]	25 [20.51-30.55]	0.012	1.81	0.285
**18:0 LPA (ng/mL)**	9.35 [8.02-10.91]	11.40 [8.95-14.52]	1.826	1.81	0.181
**18:1 LPA (ng/mL)**	10.76 [9.52-12.19]	10.86 [8.95-13.21]	0.006	1.81	0.938
**18:2 LPA (ng/mL)**	64.41 [54.95-75.50]	72.11 [56.23-92.68]	0.567	1.81	0.454
**20:4 LPA (ng/mL)**	29.78 [26.30-33.73]	30.61 [25.17-37.24]	0.055	1.81	0.816

[1]*Two-way ANCOVA was performed using lifetime CUD and sex as factors and controlling for age and BMI as covariates. Abbreviations: LPA= lysophosphatidic acid; df = degree of freedom.*

Table S10: Plasma concentrations of LPA species grouped according to use of psychiatric medication in CUD patients.

VARIABLE	No psychiatric medication (N=32)	Psychiatric medication (N=55)	Statistics[1]		
	Mean [95%CI]	Mean [95%CI]	F-value	df	*p*-value
**Total LPA (ng/mL)**	129.72 [102.57-164.44]	153.46 [137.40-171.40]	1.646	1.87	0.203
**16:0 LPA (ng/mL)**	21.73 [17.18-27.48]]	25.29 [22.69-28.18]	1.352	1.87	0.248
**18:0 LPA (ng/mL)**	9.28 [6.55-13.18]	10.02 [8.51-11.80]	0.153	1.87	0.697
**18:1 LPA (ng/mL)**	9.28 [7.41-11.61]	11.09 [10-12.33]	2.074	1.87	0.154
**18:2 LPA (ng/mL)**	55.08 [41.11-73.96]	70.46 [61.51-80.91]	2.284	1.87	0.135
**20:4 LPA (ng/mL)**	30.41 [23.28-39.71]	31.69 [27.98-35.81]	0.074	1.87	0.786

[1]*Two-way ANCOVA was performed using lifetime CUD and sex as factors and controlling for age and BMI as covariates. Abbreviations: LPA= lysophosphatidic acid; df = degree of freedom.*

Table S11: Plasma concentrations of LPA species grouped according to use of anxiolytic medication in CUD patients.

Variables	No anxiolytic medication (N=49)	Anxiolytic medication (N=37)	Statistics[1]		
	Mean [95%CI]	Mean [95%CI]	F-value	df	*p*-value
**Total LPA (ng/mL)**	144.54 [125.60-166.72]	152.75 [132.73-175.79]	0.276	1.86	0.601
**16:0 LPA (ng/mL)**	25.06 [21.78-28.91]	24.10 [20.94-27.73]	0.159	1.86	0.691
**18:0 LPA (ng/mL)**	10.09 [8.20-12.44]	9.77 [7.94-12.02]	0.050	1.86	0.823
**18:1 LPA (ng/mL)**	10 [8.77-11.43]	11.53 [10.12-13.18]	2.227	1.86	0.140
**18:2 LPA (ng/mL)**	62.23 [52.12-74.30]	71.78 [60.11-85.70]	1.249	1.86	0.267
**20:4 LPA (ng/mL)**	32.35 [27.60-38.01]	30.55 [26.06-35.73]	0.269	1.86	0.606

[1]*Two-way ANCOVA was performed using lifetime CUD and sex as factors and controlling for age and BMI as covariates. Abbreviations: LPA= lysophosphatidic acid; df = degree of freedom.*

Table S12. Correlation analysis between concentrations of LPA species and cortisol (N=34) and tryptophan metabolites (N=21) in CUD patients.

Variables	Cortisol and tryptophan metabolites					
	Cortisol (ng/mL)	TRP (pmol/mL)	KYN (pmol/mL)	KYNA (pmol/mL)	QUIN (pmol/mL)	5-HT (pmol/mL)
	Rho (*p*-value)	Rho (*p*-value)	Rho (*p*-value)	Rho (*p*-value)	Rho (*p*-value)	Rho (*p*-value)
**Total LPA (ng/mL)**	-0.178 (0.329)	0.115 (0.639)	0.202 (0.408)	**0.668 (0.002)**	0.191 (0.6222)	0.059 (0.809)
**16:0 LPA (ng/mL)**	-0.199 (0.275)	-0.076 (0.757)	0.253 (0.295)	**0.712 (<0.001)**	0.108 (0.783)	0.048 (0.846)
**18:0 LPA (ng/mL)**	-0.213 (0.242)	0.342 (0.152)	-0.059 (0.809)	0.414 (0.078)	-0.002 (0.997)	0.311 (0.195)
**18:1 LPA (ng/mL)**	-0.079 (0.667)	**0.519 (0.023)**	-0.040 (0.872)	0.407 (0.084)	0.549 (0.126)	-0.051 (0.837)
**18:2 LPA (ng/mL)**	-0.205 (0.261)	0.147 (0.548)	0.177 (0.469)	**0.609 (<0.006)**	0.156 (0.689)	-0.030 (0.904)
**20:4 LPA (ng/mL)**	-0.221 (0.223)	0.355 (0.136)	-0.020 (0.937)	0.343 (0.151)	0.547 (0.127)	-0.163 (0.505)

Bold values are statistically significant for p<0.05.

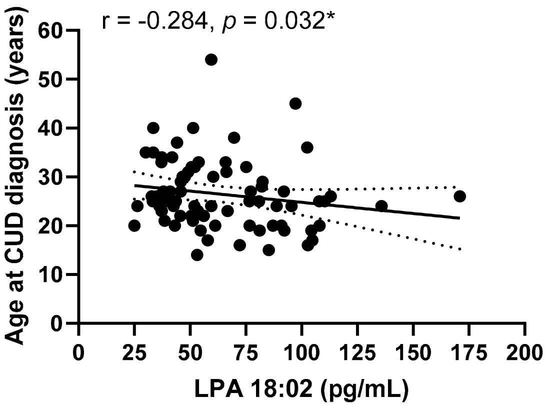

Figure S1. Correlation analysis between plasma concentrations of LPA 18:2 (pg/mL) specie and age at CUD diagnosis (years) controlled by age and BMI. Dots are individual values. (r) partial correlation coefficient; (p) p value for statistical significance.
